# Analysis of the effects of low-level laser therapy on muscle fatigue of the biceps brachii muscle of healthy individuals and spastic individuals: Study protocol for a single-center, randomized, double-blind, and controlled clinical trial

**DOI:** 10.1097/MD.0000000000017166

**Published:** 2019-09-27

**Authors:** Bruno Procopio da Silva, Gabriela Aparecida da Silveira Souza, Alexandre Alves do Nascimento Filho, Ana Paula Pinto, Carolina Lobo Guimarães, Aline Priscila Campos Pereira, Marcele Florêncio das Neves, Patrícia Sardinha Leonardo Lopes Martins, Fernanda Pupio Silva Lima, Rodrigo Alvaro Brandão Lopes-Martins, Mário Oliveira Lima

**Affiliations:** a Laboratório de Engenharia de Reabilitação Sensório Motora; b Laboratório de Biofotônica e Terapêutica Experimental, Instituto de Pesquisa e Desenvolvimento, Universidade do Vale do Paraíba, São José dos Campos, São Paulo, Brazil.

**Keywords:** infrared thermography, muscular fatigue, photobiomodulation, spasticity, stroke

## Abstract

**Background:**

Muscular fatigue is caused by biochemical alterations that modify the mechanics of muscle contraction, resulting in negative changes in the performance of the contraction. Several resources are studied to mitigate this situation among which we can cite low-level laser therapy (LLLT). The effects of LLLT are being studied in healthy subjects with fibromyalgia and who are athletes, and currently the studies are being performed in spastic muscles with poststroke individuals. The aim will be to evaluate the effects of LLLT on the fatigue of the biceps brachii muscle of healthy individuals and individuals with spastic hemiparesis.

**Methods:**

A cross-sectional, comparative, randomized, placebo, double-blind clinical trial will be divided into 2 phases: phase I shall consist of 30 healthy subjects and phase II of 30 poststroke individuals. The study will consist of 3 groups (control group, placebo group, and LLLT group), and all individuals will pass through all groups, following the randomization criteria. The protocol consists of the application of LLLT in the biceps brachii muscle on the dominant side in healthy individuals and in the hemiparetic side of poststroke individuals, and, subsequently, 3 maximal isometric voluntary contractions (MIVCs) will be performed for 50 seconds in the dynamometer, with an interval of 50 seconds between them. Pain intensity will be evaluated by means of the visual analog scale, and the myoelectric activity by means of surface electromyography associated with the evaluation of muscular strength by means of the dynamometer. The local temperature will be evaluated by infrared thermography and blood lactate concentration through the lactimeter, which will be measured at 4 different times, before the application of the laser (basal), and 3, 15, and 25 minutes after the MIVC.

## Abstract 2

Supplemental Digital Content is available in the text

## 1 Introduction

The literature describes muscle fatigue as muscle inability to maintain the strength and potency of its contractility, resulting in decreased strength, impaired motor control, and, consequently, leading to pain.^[1,2]^

Early muscular fatigue negatively influences the performance of targeted exercises, whether these exercises are performed by people with fibromyalgia, chronic obstructive pulmonary diseases, chronic fatigue, healthy elderly, athletes, and individuals presenting spasticity.^[3–8]^

Several biochemical events are related to the process of peripheral fatigue, resulting in alterations that may impair the mechanism of muscular contraction. These alterations may be triggered by modifications of creatine phosphate, glycogen, and lactic acid.^[1]^

Lactic acid diluted in body fluids is named “lactate,” and lactic acid is known as a by product of anerobic glycolysis observed in high-intensity and short-duration exercises. When not eliminated, the lactic acid dissociates and thus is converted into lactate, which causes the accumulation of H+ ions leading to muscular acidosis. With the acid concentration in the muscle there are alterations in the permeability of the sarcoplasmic reticulum, promoting the displacement of the calcium of the intramuscular environment, which directly interferes in the coupling of the crossed bridges of actin and myosin, compromising the contraction force. Moreover, the influence of the H+ ions promotes the state of exhaustion, interrupting the breakdown of glycogen and influencing the levels of ATP.^[1,9]^

Currently, changes in the epidemiological profile of global pathologies are occurring, because it is decreasing the rates of communicable diseases and increasing the rate of noncommunicable diseases, and 1 of the main factors of this inversion is stroke. A study carried out by the Global Burden of Disease, Injuries, and Risk Factors demonstrated that vascular diseases are the leading causes of death in the world and strokes are in second place. In addition, strokes are the leading cause of morbidity in the world population. In the United States, of the 795,000 new cases, 26% present difficulties in daily activities and 50% have reduced mobility.^[10]^

One of the consequences of a stroke is spasticity, characterized by the increase in muscle tone, which is velocity-dependent and is due to the hyperexcitability of the stretch reflex. Spasticity is responsible for triggering changes in sensory, proprioceptive, and musculoskeletal systems, for example, the reduction of blood circulation between muscle fibers.^[11–14]^

Phototherapy is an innovative and noninvasive alternative in the treatment and/or prevention of exercise-induced muscle fatigue, tissue injury, and in analyzing the processes involving mitochondrial function.^[2,15]^ In the literature, several studies have shown that the use of low-level laser therapy (LLLT) has positive effects on muscle fatigue attenuation and postexercise muscle recovery, when therapy is applied to before exercising.^[15–19]^

Considering the reports of studies on the benefits of using LLLT in the intact musculoskeletal system, it is believed that it may be of great value to use this resource in the treatment of the spastic muscle. dos Reis et al^[20]^ initiated a study that innovated the field of research with LLLT, applying it to the spastic musculature of knee extensors, observing a positive effect on the reduction of blood lactate, increased time preceding fatigue, and greater generation of muscular torque.

After this summary, it is believed that more studies, aimed at better understanding the effects of LLLT on the intact and spastic musculature, will be of great value in the area of motor sensory rehabilitation.

Therefore, the aim of this study is to evaluate the effects of LLLT in relation to muscular fatigue of the biceps brachii muscle of healthy individuals and spastic individuals.

## 2 Methods

### 2.1 Ethics

The project was approved by the Ethics and Research Committee of the Universidade do Vale do Paraíba (CAAE 94812618.8.0000.5503) on October 3, 2018, and subsequently registered in ClinicalTrials.gov (NCT03753984). All participants agreed and signed the free and informed consent term.

### 2.2 Study design

This is a cross-sectional, comparative, randomized, placebo, and double-blind clinical trial. Thirty healthy individuals and 30 individuals with after effects of spastic hemiparesis of both sexes will be recruited, totaling 60 individuals. The study will be divided into 2 phases, with phase I being performed with healthy individuals and phase II performed with individuals with spastic hemiparesis. The study will be carried out at the Laboratório de Engenharia e Reabilitação Sensório Motora, belonging to the Instituto de Pesquisa & Desenvolvimento of Universidade do Vale do Paraíba (Brazil).

### 2.3 Groups

All study participants will go through all groups, that is, both healthy individuals (phase I) and individuals with spastic hemiparesis (phase II) will pass through the 3 groups.

The clinical trial will be performed in 3 moments, which will be described below:

1. Control group: Individuals will not undergo LLLT, and will perform the muscular fatigue induction protocol.
2. LLLT group: Individuals will be submitted to LLLT and subsequently will perform the muscular fatigue induction protocol.
3. Placebo group: Individuals will be submitted to LLLT; however, with the device turned off and subsequently will perform muscular fatigue induction protocol.

### 2.4 Volunteers

Healthy and poststroke volunteers (with spastic hemiparesis) will be contacted.

#### 2.4.1 Inclusion criteria

Below are described the inclusion criteria for phase I (healthy individuals) and for phase II (poststroke individuals):

1. Inclusion criteria for healthy volunteers (phase I):
  - Age between 20 and 80 years
  - Both sexes
  - Preserved cognition and preservation of the ability to respond to verbal stimuli
  - Display the “insufficient active” or “active” indexes on the scale of the International Physical Activity Questionnaires (IPAQ)
2. Inclusion criteria for poststroke volunteers (phase II):
  - Individuals with medical diagnosis of stroke
  - Both sexes
  - Individuals with physiotherapeutic diagnosis of spastic hemiparesis with brachii predominance
  - Preserved cognition and preservation of the ability to respond to verbal stimuli
  - Time of injury: after 12 months
  - Patients with a maximum of second degree spasticity according to the modified Ashworth scale and minimum muscle strength of 1 in the biceps brachii muscle.

#### 2.4.2 Exclusion criteria

Below is described the exclusion criteria for phase I (healthy individuals) and for phase II (poststroke individuals):

1. Exclusion criteria for healthy volunteers (phase I):
  - Having musculoskeletal impairment of the dominant upper limb
  - Practicing physical activity with weights (body building)
  - Presence of active infection and eruptions in the dominant upper limb
  - Limiting pain that precludes the performance of the evaluation protocol
  - Ingestion of analgesic and/or anti-inflammatory drugs and/or medicines containing corticosteroids or steroids
  - Classification of Fitzpatrick: phototypes V and VI
  - Presence of malignant neoplastic lesion
  - Presence of active infection and eruption at the site of electrode application
  - Hypoesthesia and/or hyperesthesia and/or anesthesia of the limb to be applied to LLLT
2. Exclusion criteria for poststroke volunteers (phase II):
  - Presence of active infection and eruptions in the dominant upper limb
  - Limiting pain that precludes the realization of the evaluation protocol
  - Ingestion of analgesic and/or anti-inflammatory drugs and/or medicines containing corticosteroids or steroids
  - Classification of Fitzpatrick: phototypes V and VI
  - Presence of malignant neoplastic lesion
  - Presence of active infection and eruption at the site of electrode application
  - Hypoesthesia and/or hyperesthesia and/or anesthesia of the limb to be treated
  - Muscular contractures and articular deformities
  - Uncontrolled arterial hypertension
  - Individuals who present other neurological and/or orthopedic alterations associated
  - Wernick or Broca aphasia

### 2.5 Randomization and blinding

All individuals (phase I and phase II) will go through all the study groups (control group, placebo group, and LLLT group) with a minimum interval of 7 days between them; however, all individuals will initiate the protocol in the control group.

The randomization of the sample will be performed in the second session in which the individuals will be divided into two classes: class A and class B. Individuals who draw class A will start in the placebo group and finalize in the LLLT group, whereas individuals who draw class B will start in the LLLT group and subsequently perform the placebo group, making it a cross study.

**Figure 1:**
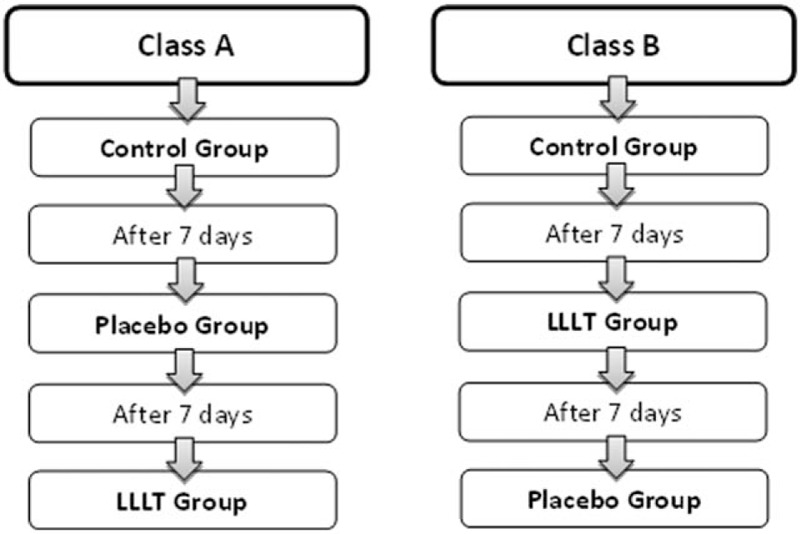
Flowchart indicating sample randomization.

Researcher 1 will be responsible for the instrumentation of the LLLT apparatus, whereas researcher 2 will be responsible for analyzing the data. Only researcher 1 will know in which group the individual will be allocated, and the type of treatment used will be hidden from researcher 2 and the participant. Figure 1 shows the flowchart indicating the randomization of the sample.

### 2.6 Evaluation methods

Below is described the evaluation methods that will be used in the study.

#### 2.6.1 Surface electromyography

The myoelectric signals will be collected by an electromyographer (model EMG 832C—WIFI, made by EMG System Brasil, Brazil) consisting of 8 channels with a 12-bit resolution A/D converter (Analog-digital converter), with a sampling frequency of 2000 Hz, Unit μv, Xmin-2000, Ymax 2000, Coef. A 0 and Coef. B 500, coupled to a computer.

The surface electrodes will be placed in pairs on the muscle surface of the biceps brachii muscle, accompanying the longitudinal direction of the muscular fibers. The location of the muscle surface will follow the recommendations of SENIAM (Surface Electro MyoGraphy for the Non-Invasive Assessment of Muscles). A gel-anointed type electrode will be positioned in the styloid process of the ulna of the upper limb contralateral to the limb that will be evaluated.

#### 2.6.2 Infrared thermography

For the collection of infrared thermography, an infrared, FLIR model S65 camera will be used, coupled to a professional photographic tripod (1.5 meters Canon), connected to a computer with the Software Thermo Cam Researcher Professional 2.10.

The camera will be positioned 1 m away from the common image capture location of 45°, following the standardization of the emissivity parameters (0.98 of the human skin), ambient temperature 22°C (±0.9°C) and relative humidity 60%.

Five images will be captured in each group (control group, placebo group, and LLLT group):

- Image 1 (basal capture) will be recorded after climatization, and in the case of the placebo group and LLLT group before the laser application;
- Image 2 (basal capture post LLLT) will be recorded after the laser application (placebo group and LLLT group) and before the maximal isometric voluntary contraction (MIVC);

Images 3, 4, and 5 will be recorded after the MIVC of the individual:

- Image 3 will be recorded 3 minutes after MIVC;
- Image 4 will be recorded 15 minutes after the MIVC;
- Image 5 will be recorded 25 minutes after the MIVC;

Thus the capture of the images will occur before the MIVC (basal), after the LLLT (basal post LLLT), and 3, 15, and 25 minutes after the MIVC.

#### 2.6.3 Dynamometry

Associated with the surface electromyography (EMG) capture, the muscle torque will be measured from the MIVC of the biceps brachii muscle for which a traction dynamometer (model DFE021115/200, EMG System do Brasil, Brazil) will be used.

The traction dynamometer will be coupled to the electromyograph. This way, the muscle strength data collection will be performed in synchronization with the myoelectric activity.

#### 2.6.4 Visual analog scale

Pain intensity will be assessed by the visual analog scale (VAS) of pain which consists of a horizontal line with a scale from 0 to 10, where 0 represents absence of pain and 10 represents the worst possible pain. In this way, the volunteer will be instructed to indicate his pain intensity before and after the induction of muscular fatigue.

#### 2.6.5 Lactate assessment

For the collection of blood lactate levels the Accutrend lacmeter by Roche will be used in 4 moments: before the MIVC in the traction dynamometer (baseline measurement), and 3, 15, and 25 minutes after the MIVC.

### 2.7 Low-level laser therapy

The protocol for the application of LLLT will be performed through the continuous punctual transcutaneous direct method to radiate 16 points in the biceps brachii muscle with approximately 1 cm of distance between the points.

This will be done using a laser diode, with infrared λ = 808 nm, 0.028 cm^2^ of beam area, 100 mW power, a power density of 3.5 W/cm^2^, 4 Joules/point of energy, and an energy density of 142.8 J/cm^2^.

During the LLLT application all biosafety measures will be adopted, that is, all those present at the collection site will be properly equipped with safety goggles suitable to the wavelength used and the light pointer will be covered with a plastic protective film.

### 2.8 Outcomes

Because this is a cross-sectional study, the outcomes will be obtained by comparing the data obtained between the groups with the aim of observing the behavior of muscular fatigue in relation to the use of LLLT, by means of the above-mentioned evaluation methods.

### 2.9 Study protocol

Initially, an assessment of the individual will be carried out to analyze whether it fits into the study in question, considering the inclusion and exclusion factors already described. The individuals of the poststroke group will undergo the subjective spasticity assessment by three physiotherapists (double blind), through the modified Ashworth scale and manual evaluation of muscle strength. The term of free and clarified consent will be presented (Appendix A).

All volunteers will start in the control group and then on the second contact they undergo the study randomization as described above. The experiment procedures will be standardized in the 3 study groups according to the following sequence of events: placement of the surface electrodes in the biceps brachii muscle, climatization of the individual for 10 minutes during which the VAS and initial blood lactate measurement (baseline measurement) will be made, and after 10 minutes of air conditioning image 1 will be captured by means of infrared thermography.

To perform the muscular fatigue induction protocol of the biceps brachii muscle, the individual will be positioned at the Scott bench with the elbow at 45°. An inelastic band will have 1 of its fixed extremities in the traction dynamometer and the other end positioned on the forearm of the volunteer to perform the MIVC protocol of the brachii biceps muscle.

The protocol consists of 3 MIVCs during 50 uninterrupted seconds with an interval of 50 seconds between each contraction. During the contractions the myoelectric activity and muscular strength will be collected synchronically.

The assessments after the MIVC will be performed at three distinct moments (3, 15, and 25 minutes) following the same sequence of events: capture of the image by means of infrared thermography (images 3, 4, and 5) and blood lactate measurement.

The placebo group and the LLLT group will first undergo the climatization for 10 minutes, VAS application, blood lactate measurement (baseline measurement), image 1 capture by means of infrared thermography and LLLT application. After 3 minutes, the capture of image 2 will be performed by infrared thermography, and the surface electrodes will be positioned in the biceps brachii muscle. Subsequent events were the same as above.

**Figure 2:**
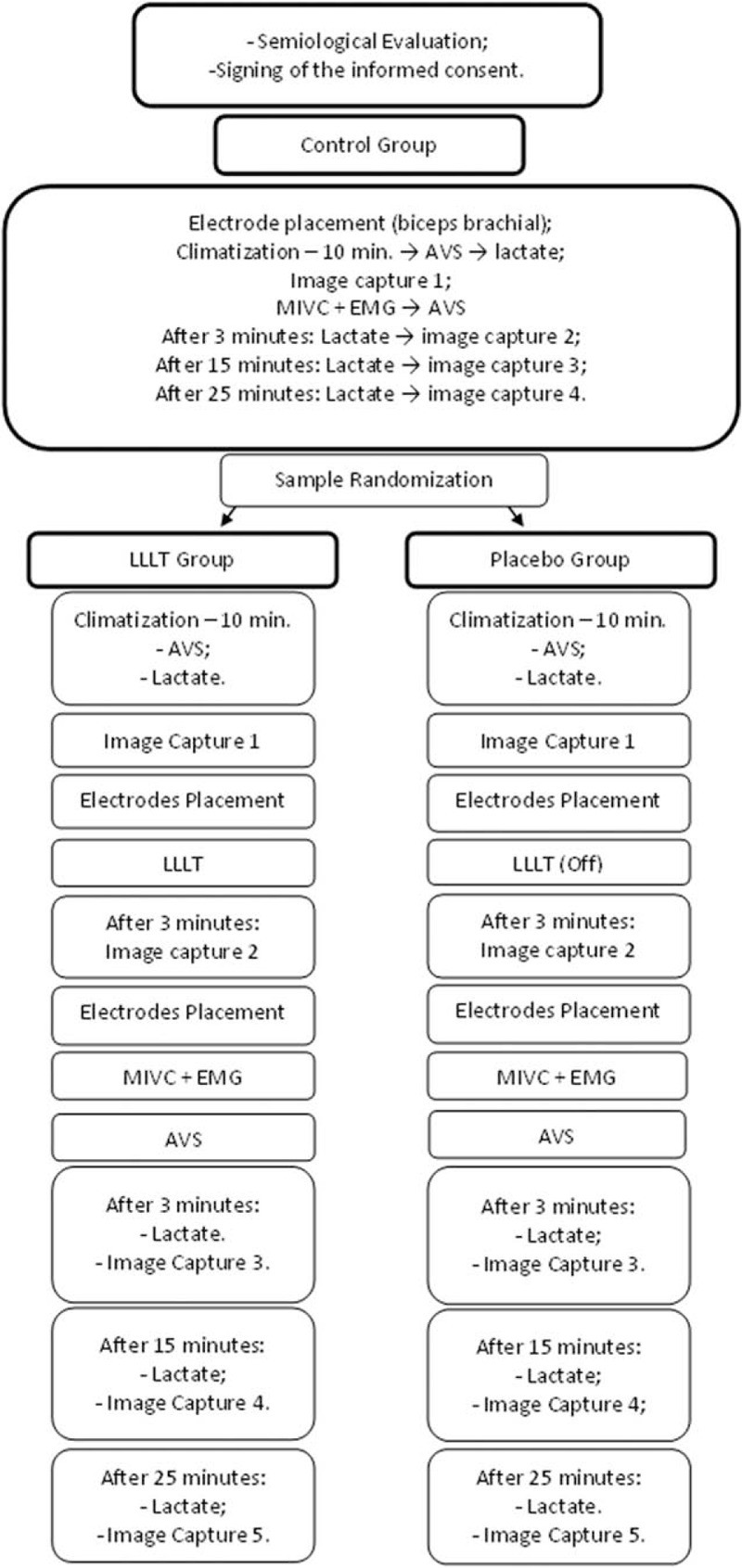
Flowchart demonstrating the study protocol. EMG = surface electromyography, LLLT = low-level laser therapy, MIVC = maximal isometric voluntary contraction, VAS = visual analog scale.

For a better visualization of the sequence of events of the study a flowchart was made as shown in Fig. 2.

### 2.10 Statistical analysis

To assess the effects of LLLT, normality tests will be applied which allow the selection of a more adequate statistical test to perform the intra and intergroup comparisons. Statistical significance will be adopted as *P* < 0.05. The data and results will be presented through tables and graphs. Scientific Data Analysis and Visualization (SciDAVis, USA) will be the software of choice.

## 3 Discussion

There are few studies found in the literature regarding the applicability of LLLT in the spastic muscles associated with muscular fatigue. However, this framework is of real relevance in the rehabilitation process.^[12–14]^

When searching the PubMed database using the keywords “laser and fatigue” associated with the filter “ClinicalTrial” 90 scientific articles were found, of which only 2.23% (2 papers) were directed to spastic musculature. Using the combination of the keywords “laser; Fatigue Spastic muscle” 2 more papers were found, totaling 4 scientific articles addressing the use of laser in muscular fatigue of spastic muscles.

To perform this study, evaluation methods that can prove the real effects of LLLT in a synchronous and simultaneous manner will be used. These are contemporary methods such as infrared thermography associated with surface electromyography (EMG), dynamometry, and lactate analysis, which will present results in relation to the actual effect of LLLT on muscular fatigue behavior.

dos Reis et al^[20]^ conducted a pioneering study where LLLT was applied to the spastic musculature of knee extensors in individuals after a stroke. The results showed a reduction in blood lactate, increased time of onset of muscle fatigue, and increased muscle torque.

Using methods of evaluation and induction of muscular fatigue similar to the previous study, Neves et al^[8]^ conducted a study with 15 volunteers with spasticity after a stroke. In this study, muscle endurance tests were performed in the Isokinetic dynamometer where the time of onset of muscle fatigue and peak torque was evaluated. According to the results of the study, it was observed that when applying LLLT in the spastic musculature before the induction of muscular fatigue, there was an increase in the time of onset of muscular fatigue and increased peak torque.

The other 2 articles found in the literature were performed by Santos et al (2016)^[21]^ and Santos et al. (2017),^[22]^ in which they applied LLLT in the masseter muscle of children with spasticity due to cerebral palsy. In the first article,^[21]^ the amplitude of the mouth opening and the analysis of the tonus of the masseter muscle were evaluated over 3 weeks of treatment, and an increase in the amplitude of the buccal opening and decrease of muscle tone were found. The second clinical study^[22]^ aimed at evaluating the thickness of the masseter muscle by means of ultrasonography, mouth opening amplitude, and the impact on oral health-related quality of life after 6 applications of LLLT showed an increase in the thickness of the masseter and increase in oral amplitude, and in relation to the questionnaire of quality of life, there was an improvement in all domains, except for well-being.

## 4 Conclusions

Thus, the importance of the study in question is justified, aiming to analyze the effects of LLLT in relation to muscular fatigue to improve the performance of the individual during the rehabilitation process, bringing positive effects on quality of life and functional quality. In addition, it aims to analyze the effects of LLLT in relation to muscular fatigue of healthy individuals and compare with the effects triggered in the spastic muscles.

## Author contributions

**Conceptualization:** Ana Paula Pinto, Aline Priscila Campos Pereira, Fernanda Púpio Silva Lima, Rodrigo Alvaro Brandão Lopes Martins, Mário Oliveira Lima.

**Data curation:** Gabriela Aparecida da Silveira Souza, Alexandre Alves do Nascimento Filho, Ana Paula Pinto, Carolina Lobo Guimarães, Patrícia Sardinha Lopes Martins Leonardo.

**Formal analysis:** Bruno Procópio Da Silva, Gabriela Aparecida da Silveira Souza, Alexandre Alves do Nascimento Filho, Carolina Lobo Guimarães, Marcele Florêncio Das Neves, Patrícia Sardinha Lopes Martins Leonardo, Fernanda Púpio Silva Lima, Mário Oliveira Lima.

**Funding acquisition:** Mário Oliveira Lima.

**Investigation:** Bruno Procópio Da Silva, Gabriela Aparecida da Silveira Souza, Alexandre Alves do Nascimento Filho, Patrícia Sardinha Lopes Martins Leonardo.

**Methodology:** Alexandre Alves do Nascimento Filho, Ana Paula Pinto, Carolina Lobo Guimarães, Aline Priscila Campos Pereira, Marcele Florêncio Das Neves.

**Project administration:** Rodrigo Alvaro Brandão Lopes Martins.

**Resources:** Bruno Procópio Da Silva.

**Supervision:** Fernanda Púpio Silva Lima, Rodrigo Alvaro Brandão Lopes Martins, Mário Oliveira Lima.

**Validation:** Bruno Procópio Da Silva, Gabriela Aparecida da Silveira Souza, Ana Paula Pinto.

**Visualization:** Bruno Procópio Da Silva, Marcele Florêncio Das Neves, Patrícia Sardinha Lopes Martins Leonardo.

**Writing – original draft:** Bruno Procópio Da Silva, Gabriela Aparecida da Silveira Souza, Fernanda Púpio Silva Lima, Rodrigo Alvaro Brandão Lopes Martins, Mário Oliveira Lima.

**Writing – review & editing:** Rodrigo Alvaro Brandão Lopes Martins, Mário Oliveira Lima.

## Supplementary Material

Supplemental Digital Content
