# Supplementary material for: Analysis of the effects of low-level laser therapy on muscle fatigue of the biceps brachii muscle of healthy individuals and spastic individuals: Study protocol for a single-center, randomized, double-blind, and controlled clinical trial
Source: Medicine (Baltimore). 2019 Sep 27;98(39):e17166. doi: 10.1097/MD.0000000000017166 (PMC6775413; doi:10.1097/MD.0000000000017166)
Supplement: Supplemental Digital Content [file medi-98-e17166-s001.docx]

**Free and informed consent term**

**Identification info**

**Project Title**: Analysis of the effects of low level laser therapy on muscle fatigue of the biceps brachii muscle of healthy individuals and spastic individuals

**Principal Researcher:** Physiotherapist Gabriela Aparecida da Silveira Souza.

**Executive team:** Gabriela Aparecida da Silveira Souza, Mário Oliveira Lima, Rodrigo Álvaro Brandão Lopes Martins, Fernanda Pupio Silva Lima.

**Institution to which the Researcher in charge belongs:** University of Vale do Paraíba, Research and Development Institute.

**Telephones for contact:** Reception of the physiotherapy clinic University of Vale do Paraíba: (12) 3947-1086

**Researcher's cell phone:** (12) 99723 - 2806

**Ethics and Research Committee of University of Vale do Paraíba:** (12) 3947-1111

**Name:** _______________________________________________________________

**Age:** _______________________ **R.G.:** ___________________________________

You ________________________________________________ are invited to participate in the master's research project entitled: “Analysis of the effects of low intensity laser therapy on muscle fatigue of the biceps brachii muscle of healthy and spastic individuals: evaluation by infrared thermography, surface electromyography, isokinetic dynamometer and lactimeter ”by researcher Gabriela Aparecida da Silveira Souza.

This document (Free and Informed Consent Form) consists of two identical copies, one copy of which will be with the researcher in charge and the other copy will be of the participant.

The aim of this study was to evaluate the effect of laser on tiredness in the arm muscles (biceps brachii and triceps brachii) of healthy participants and stroke. Initially an evaluation will be performed, which will explain all the phases of the study, and if accepted, the participant will authorize their participation through this document. The participant is expected to attend the Biodynamics Laboratory at the University of Vale do Paraíba (UNIVAP), located at Av. Shishima Hifumi, 2911, Urbanova, blocks 7, at three different times, with an interval of 7 days between them. When arriving at the sector it will be necessary for the participant to carry out the acclimatization, he will be seated for 10 minutes in a climate room at 22 ° C. During the tests will be evaluated muscle contraction (electromyography) at the moment of maximum arm muscle strength (biceps and triceps brachii), muscle strength, evaluation of muscle pain, evaluation of images indicating body temperature (thermography) and collection of a substance in the body that indicates muscle fatigue (blood lactate) through a blood droplet following a small puncture in the fingertip, with the aid of a disposable needle.

The collection of the substance that indicates muscle fatigue will be performed at four times, before the muscle fatigue test, and at 3, 15 and 25 minutes after the muscle fatigue test. All hygiene and safety measures will be used throughout the test, including the use of disposable gloves by the researcher. For the maximum strength test the participant will be positioned on a piece of equipment and asked to make the elbow bend movement (elbow flexion) for 50 seconds.

The study will consist of three (3) phases, with an interval of seven (7) days between them, and all participants will perform the three (3), and in the first phase will be performed the protocol already described above, however without laser therapy application, called Control Group. The other two phases will be composed by the Placebo Group, in which the participant will receive the simulation of the Laser application with the device turned off and the Laser Therapy Group, in which the participant will receive the Laser application with the device turned on. It will not be revealed to the participant which group they will be in (Placebo Group or Laser Therapy Group), however this will not harm the participant, it is only necessary to evaluate the real effects of Laser Therapy.

Placebo is a sham treatment, used to compare with a new therapy proposal, to see if the new treatment has a real effect. It is already proven that only by offering a therapy to an individual, it is possible that the body itself has a positive response, even if the treatment is not true (simulation of a therapy), generating the placebo effect. Therefore, in this study the placebo group will receive the simulation of laser treatment, in which the device will be turned off (no light will be offered) in order to compare with the laser therapy group, in which the device will be turned on, offering the possible benefits. of laser therapy. In the Laser Therapy Group, the arm will be evaluated by a professional with experience in the application of the technique, using a laser commercially available from Clean Line®.

Research risks are related to the body's responses when exposed to physical exercise, such as muscle pain or discomfort and physical tiredness while performing the test. This way, the participant will be free to interrupt the test at any time if he / she feels any discomfort, pain or exhaustion.

If the participant presents pain exacerbation or any major discomfort caused by the protocol proposed by the study, it will be interrupted. In case of persistent or exacerbated pain, the participant will be instructed to use Tylenol® as a standard analgesic, which does not require a prescription (OTC), as it has no anti-inflammatory effect and only analgesic effect.

In case of intercurrence, the participant will be rescued by the SAMU ambulance of the city of São José dos Campos and sent to the municipal hospital Dr. Jose de Carvalho Florence of São José dos Campos, namely Vila Industrial Hospital, where he will receive medical assistance by SUS. Researchers will provide full assistance to all participants during the study period regarding the complications caused. This will be recorded in the minutes and those responsible for the study will notify the ethics committee.

The expected benefits are due to the fact that Laser (Low Intensity Laser Therapy) decreases the time of onset of muscle fatigue, as well as improving the movement of the paralyzed arm, by increasing muscle electrical activity and improving muscle strength which will result in improvement of functionality and quality of life of the individual. Whenever necessary, the participant may contact for information about the research project, its participation in it or other research-related matters, with the responsible researcher, executing team or at the Research Ethics Committee of the University of Vale do Vale. Paraíba (UNIVAP) by telephone (12) 3947-1111, or in person at Av. Shishima Hifumi, 2911, Urbanova, block 11- Institute for Research and Development II, room 19, Monday to Friday, 8:00 am-12pm : 00h and 13: 00h-17: 00h.

Please be aware of this term that by agreeing to participate in this research the participant may withdraw at any time prior to or during the study without penalty, harm to their health or loss of any benefit for their follow-up or suffer any sanctions or constraints.

The data obtained from the participant may not be used for purposes other than scientific research, being disclosed without identification only in scientific and academic media (publication of scientific articles, presentations in congresses, symposiums and classes), and will be confidential, confidential and privacy in accordance with the ethical standards for research involving human beings, of the National Research Ethics Commission (CONEP) of the National Health Council, of the Ministry of Health.

For the recruitment of healthy participants, staff and students of the Faculty of Health Sciences and patient companions of the Center for Supervised Practice (CPS) will be contacted and invited, where they will be personally addressed by the project researchers. Individuals after stroke will be recruited from the waiting list of the school clinic - CPS located at the University of Vale do Paraíba - UNIVAP.

I, ___________________________________________________________, RG nº _____________________ was informed and agree to participate as a patient in the research project described above.

São José dos Campos, _____ de ____________ de _______.

____________________________ ______________________________

Name and Signature Name and signature of person responsible for obtain consent

___________________________ ___________________________

Witness Witness
